# Bisoprolol protects myocardium cells against ischemia/reperfusion injury by attenuating unfolded protein response in rats

**DOI:** 10.1038/s41598-017-12366-8

**Published:** 2017-09-19

**Authors:** Chengcheng Zhang, Songqing He, Yanming Li, Feng Li, Zhengbing Liu, Jing Liu, Jianbin Gong

**Affiliations:** Department of Cardiology, Jinling Hospital, Nanjing University School of Medicine, Nanjing, Jiangsu China

## Abstract

Bisoprolol (B) exerts potential cardioprotective effects against myocardial ischemia/reperfusion (I/R) injury. Unfolded protein response (UPR) attenuates I/R injury induced apoptosis by reducing oxidative damage and inflammation response. The current study investigated whether the protective effects of bisoprolol resulted from modulating UPR and anti-inflammatory during myocardial I/R condition and elucidated its potential mechanisms. Sprague-Dawley rats were treated with B in the absence or presence of the injected UPR activator dithiothreitol (DTT) and then subjected to myocardial I/R surgery. *In vitro*, cultured H9C2 cells were pretreated with B or DTT and then subjected to simulate ischemia reperfusion (SIR) operation. Bisoprolol conferred cardioprotective effects by improving postischemic cardiac function, decreasing infarct size, reducing apoptotic index, diminishing serum creatine kinase and lactate dehydrogenase levels, suppressing TNF-α and IL-6 secretion, inhibiting UPR signal pathways and downregulating caspase-12 and caspase-3 expressions. Consistently, B conferred similar antioxidative and anti-inflammatory effects against SIR injury in cultured H9C2 cardiomyocytes. Pretreatment with DTT or C/EBP homologous protein (CHOP) overexpression mediated by lentivirus administration both abolished these effects. In summary, our results demonstrate that Bisoprolol protects myocardium cells against ischemia/reperfusion injury partly by attenuating unfolded protein response.

## Introduction

Ischemic heart disease including acute coronary syndrome remains the leading cause of mortality and disability worldwide^1^. The most efficient treatment of ischemic cardiovascular disease is timely reperfusion, including primary percutaneous coronary intervention and thrombolytic therapy. However, reperfusion itself results in a further cardiomyocyte damage, which is commonly referred to myocardial ischemia/reperfusion (I/R) injury^2,3^. It has been shown that myocardial I/R injury triggers many distinct and overlapped cell signaling pathways, and finally decide the cell to survival or death^4^.

The endoplasmic reticulum (ER) is a multifunctional cellular organelle, which is recognized as the principal site of protein biosynthesis and folding, lipid biosynthesis, cell homeostasis, calcium homeostasis and apoptosis^5^. Myocardial I/R injury is known to result in the accumulation of unfolded proteins in the ER, and the unfolded protein response (UPR) is activate to deal with this disadvantageous situation^6^. There were studies demonstrated that during myocardial I/R injury in the myocardium ATF6 branch of UPR is activated and help to reduce the myocardial I/R injury^7,8^. The C/EBP homologous protein (CHOP) and caspase-12 were involved in ER stress-induced apoptosis, which were confirmed in many studies^9–11^.

Actually, UPR acts as a sentinel of protein folding in the ER, restoring the ER homeostasis, while the cell death or apoptosis will ensue with the UPR persistent^12^. In the eukaryotic cells, UPR is characterized by the activation of three ER transmembrane effector proteins: transcription factor-6 (ATF-6), inositol requiring enzyme 1 (IRE1), and PKR-like ER kinase (PERK)^13^, which mediated three branches signal pathway of UPR. Glucose-regulated protein 78 (GRP78), is one of the best characterized UPR target gene in ER. Upon ER stress, sequestration of GRP78 with unfolded proteins will activate the three UPR sensors^14^. However, to date, whether myocardial I/R injury activate all the three branches of UPR pathway is still largely unknown. Activated IRE1 pathway splices the mRNA of a transcription factor called X-box-binding protein-1 (XBP1), removing a 26-bp nucleotide intron from the full-length XBP1 mRNA that creates a translational frame shift leading to the expression of a spliced XBP1 (sXPB1)^15,16^. The molecular of sXBP1 is a highly active transcription factor for ER-resident enzymes and chaperones^15^. Activated PERK phosphorylates eukaryotic translation initiation factor 2 subunit α (eIF2α), which leads to inhibition of global protein synthesis^17^. However, phosphorylated eIF2α can also lead to an increase expression of ATF4 and CHOP^18^. When ER stress is excessive and/or prolonged, however, apoptotic signals are initiated by the UPR in the ER, including induction of CHOP, activation of Jun N-terminal kinase (JNK), and cleavage of caspase-12^19–21^.

β-adrenoceptor antagonists (β blockers) are indicated for patients with mild to moderate heart failure (New York Heart Association (NYHA) class II and III) of ischaemic or non-ischaemic aetiology^22^. Treatment with β-blockers exerts a range of beneficial effects on cardiac function and, importantly, reduces HF-related morbidity and mortality^23,24^. Importantly, it has also been reported that bisoprolol significantly reduced long-term cardiac death and myocardial infarction in high-risk patients after successful major cardiac vascular surgery^25^. However, the underlying molecular mechanism(s) responsible for this effect remains unidentified.

In the article, Bisoprolol was used as an adjuvant to attenuate the UPR induced by myocardial I/R injury. In addition, the involvement of the UPR signaling pathway and apoptosis in bisoprolol’s protective actions were also evaluated.

## Results

### Effect of exogenous bisoprolol and dithiothreitol (DTT) treatment on Sham operated rat heart

**Figure 1:**
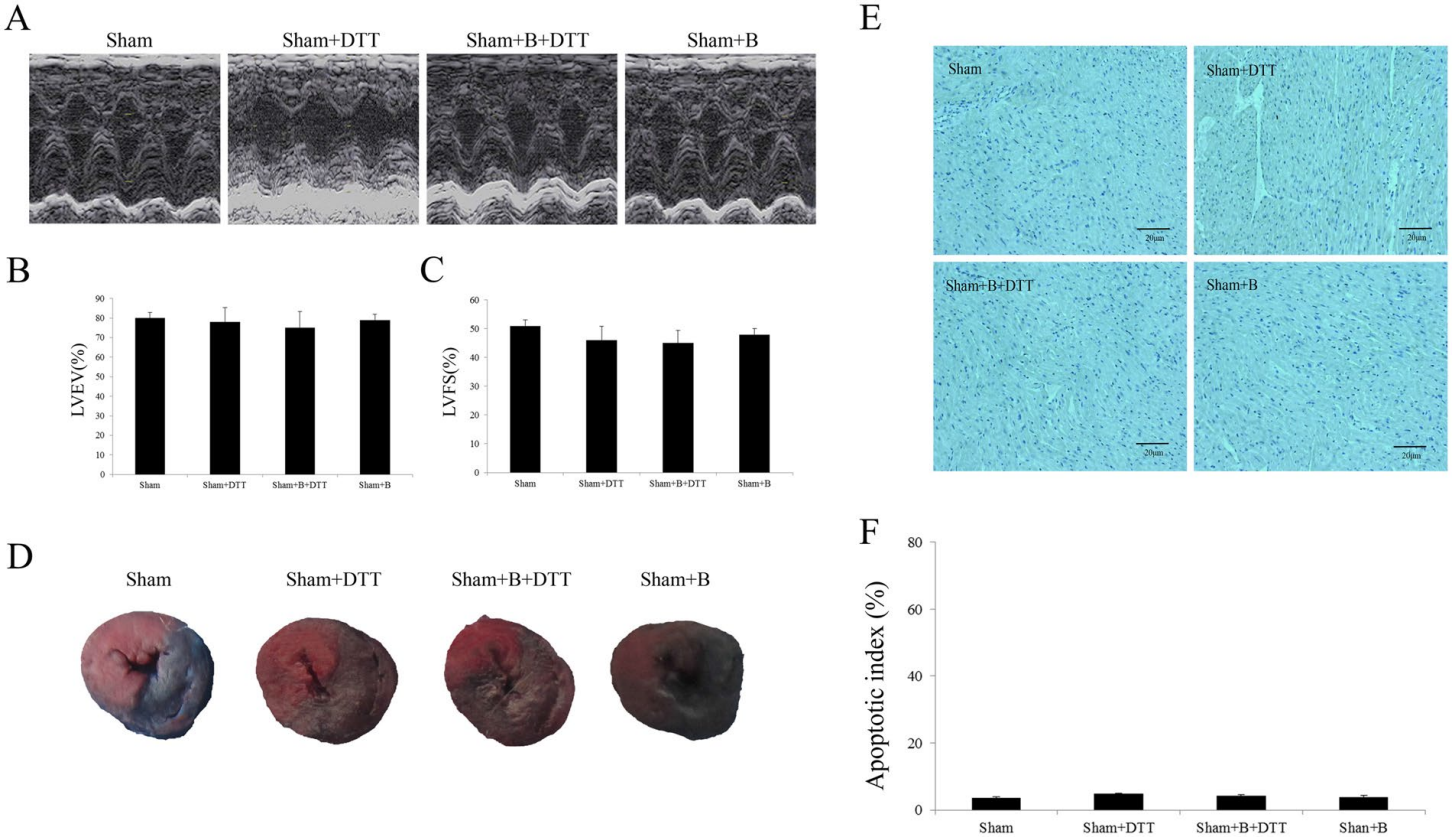
Effect of bisoprolol and DTT treatment on sham-operated rats heart. Normal SD rats pretreated with B or DTT were subjected to the Sham operation. The sham-operated animals underwent the same surgical procedures except that the suture around the LCA was not fastened. Echocardiography measurement was performed after 24 h of reperfusion. The myocardial infarct size and apoptosis index was performed after 6 h of reperfusion. (**A**) Representative images of M-mode echocardiographic. (**B**) Left ventricular ejection fraction (LVEF). (**C**) Left ventricular fractional shortening (LVFS). (**D**) Representative images of heart section. The Evans blue-stained portion (blue) indicate the nonischaemia, normal region; the TTC-stained areas (red) indicate ischemic but not infarcted tissue; and the Evans blue/TTC-unstained (negative) areas (white) indicate infarcted region. (**E**) Representative images of apoptotic cardiomyocytes by TUNEL staining. Blue staining indicates the nucleus of each cell, the dark brown staining indicates apoptotic cardiomycytes. (**F**) Percentage of TUNEL-positive nuclei. The results are expressed as the mean ± SEM. n = 6/group.

**Figure 2:**
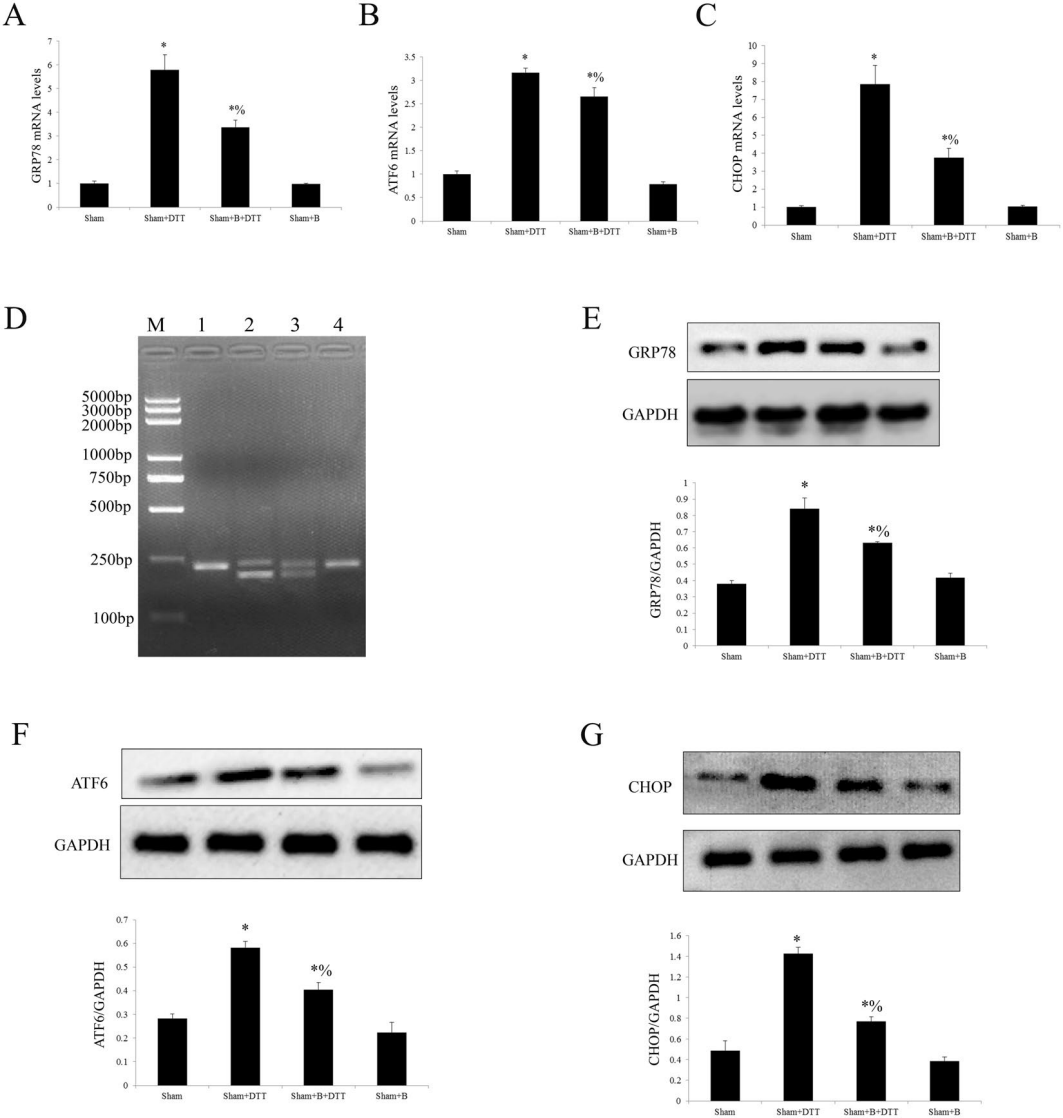
Effect of bisoprolol and DTT treatment on sham-operated rat hearts. (**A**) Analysis the transcript level of GRP78 by qRT-PCR. (**B**) Analysis the transcript level of ATF6 by qRT-PCR. (**C**) Analysis the transcript level of CHOP by qRT-PCR. (**D**) Analysis the effect of XBP1 processing by RT-PCR. (**E**) Representative Western blotting illustrating the expression of GRP78 and GAPDH and the quantitative analysis GRP78 expression level after normalization to GAPDH. (**F**) Representative Western blotting illustrating the expression of ATF6 and GAPDH and the quantitative analysis ATF6 expression level after normalization to GAPDH. (**G**) Representative Western blotting illustrating the expression of CHOP and GAPDH and the quantitative analysis CHOP expression level after normalization to GAPDH. The results are expressed as the mean ± SEM. n = 6/group. *P < 0.01 vs the sham group; % P < 0.01 vs the sham + DTT group.

Firstly, in the *in vivo* experiment, we evaluated the effect of exogenous B or DTT treatment on the sham-operated rat hearts. Compared with the sham group, neither B nor DTT treatment in Sham operated rat significantly affected the cardiac function including Left ventricular ejection fraction (LVEF) and left ventricular fractional shortening (LVFS) value (P > 0.05, Fig. 1A–C). In addition, neither the myocardial infarct size nor the myocardial apoptosis were markedly changed compared with the sham group (P > 0.05, Fig. 1D–F). Moreover, we detected the effects of B or DTT on the cellular UPR signaling pathway. As shown in Fig. 2, as a UPR activator, DTT treatment markedly actived the three branches pathway of UPR (P < 0.01), B treated alone had no significant effect (P > 0.05). While, compared with Sham + DTT group, in the Sham + B + DTT group the expression of the molecular of UPR GRP78, ATF6 and CHOP was significant decreased (Fig. 2, P < 0.01).

### Exogenous bisoprolol treatment significantly attenuated the myocardial I/R injury and UPR, where these effects can abolish by DTT co-treatment

**Figure 3:**
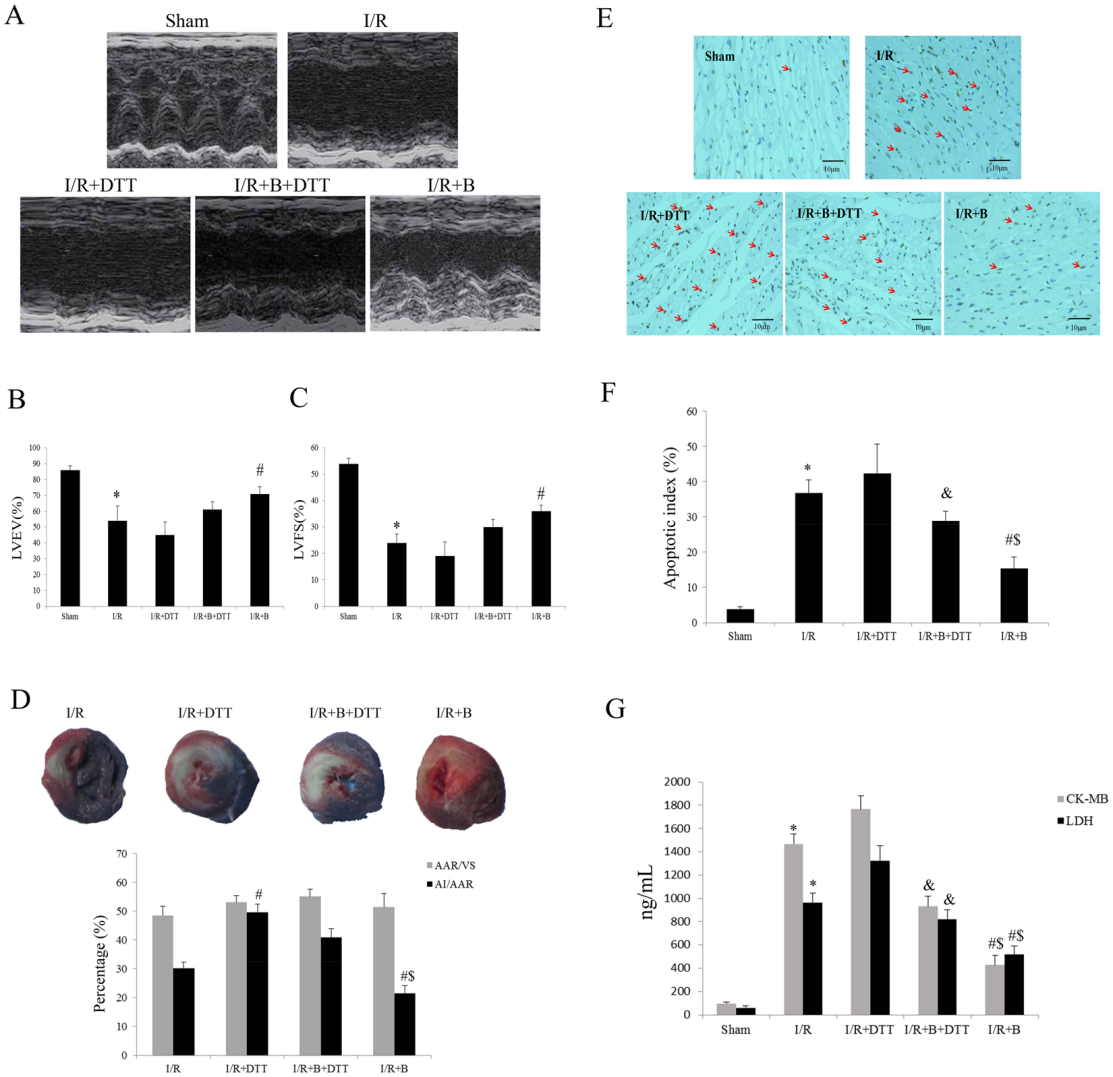
Effect of bisoprolol and DTT treatment on I/R operated rat hearts. Normal SD rats pretreated with B or DTT were subjected to the ischemic reperfusion operation. Echocardiography measurement was performed after 24 h of reperfusion. The myocardial infarct size and apoptosis index was performed after 6 h of reperfusion. (**A**) Representative images of M-mode echocardiographic. (**B**) Left ventricular ejection fraction (LVEF). (**C**) Left ventricular fractional shortening (LVFS). (**D**) Representative images of heart section. The Evans blue-stained portion (blue) indicate the nonischemic, normal region; the TTC-stained areas (red) indicate ischemic but not infarcted tissue; and the Evans blue/TTC-unstained (negative) areas (white) indicate infarcted region. (**E**) Representative images of apoptotic cardiomyocytes by TUNEL staining. Blue staining indicates the nucleus of each cell, the dark brown staining indicates apoptotic cardiomycytes. (**F**) Percentage of TUNEL-positive nuclei. (**G**) Effect of pretreat with UPR stimulator DTT and B on plasma LDH and CK-MB activities. The results are expressed as the mean ± SEM. n = 6/group. *P < 0.01 vs the sham group; ^#^P < 0.01 vs the I/R group. ^&^P < 0.01 vs the I/R + DTT group; ^$^P < 0.01 vs the I/R + B + DTT group.

*In vivo* experiment, we evaluated the effect of exogenous B or DTT treatment on the myocardial I/R-operated rat hearts. Compared with the myocardial I/R group, B treatment effectively improved the post-myocardial I/R cardiac function recovery as indicated by the increased LVEF and LVFS (P < 0.01, Fig. 3A–C). However, compared with the myocardial I/R + B group, these protective effects were blocked by DTT co-treatment (P < 0.01, Fig. 3A–C). Particularly, compared with the myocardial I/R group, treated with DTT alone aggravate the injury of heart (Fig. 3A–C). Next, we evaluated the myocardial infarct sizes. As shown in Fig. 3D, the B-treated group showed a significantly decreased myocardial infarct size (compared with the I/R group, P < 0.01), while the DTT treatment significantly blocked this effect (compared with the I/R + B group, P < 0.01). We also find that the infarct size in I/R + DTT is bigger than the I/R groups (Fig. 3D). We also measured the levels of myocardial apoptosis. As shown in Fig. 3E and F, the myocardial apoptosis caused by the myocardial I/R injury was also markedly attenuated by B treatment, and this attenuation was also abolished by the DTT co-treatment. The B treatment significantly decreased the serum lactate dehydrogenase (LDH) and creatine kinase MB (CK-MB) levels, and these effects were also blocked by DTT treatment (P < 0.01, Fig. 3G).

**Figure 4:**
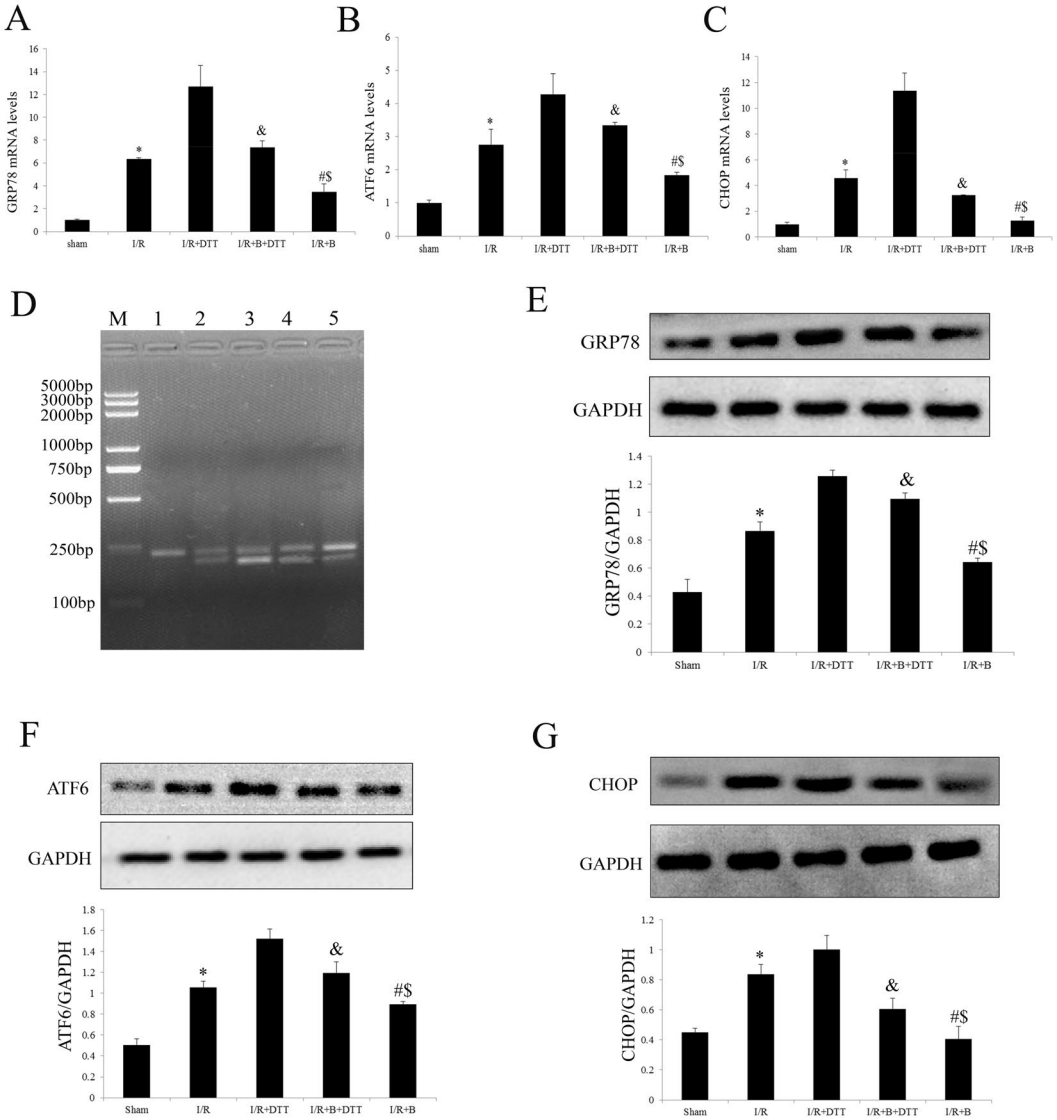
Effect of bisoprolol and DTT treatment on I/R operated rat hearts. (**A**) Analysis the transcript level of GRP78 by qRT-PCR. (**B**) Analysis the transcript level of ATF6 by qRT-PCR. (**C**) Analysis the transcript level of CHOP by qRT-PCR. (**D**) Analysis the effect of XBP1 processing by RT-PCR. (**E**) Representative Western blotting illustrating the expression of GRP78 and GAPDH and the quantitative analysis GRP78 expression level after normalization to GAPDH. (**F**) Representative Western blotting illustrating the expression of ATF6 and GAPDH and the quantitative analysis ATF6 expression level after normalization to GAPDH. (**G**) Representative Western blotting illustrating the expression of CHOP and GAPDH and the quantitative analysis CHOP expression level after normalization to GAPDH. The results are expressed as the mean ± SEM. n = 6/group. *P < 0.01 vs the sham group; ^#^P < 0.01 vs the I/R group. ^&^P < 0.01 vs the I/R + DTT group; ^$^P < 0.01 vs the I/R + B + DTT group.

To further understand the molecular mechanism of B’s cardioprotective effect, we investigated the myocardial UPR signaling pathway. Figure 4 showed that B markedly down-regulated the GRP78, ATF6 and CHOP both in transcript and protein levels in the myocardial I/R-injured hearts (compared with the I/R group, P < 0.01). The splice effect of XBP in I/R and I/R + DTT group was activated which detected by RT-PCR (Fig. 4D). However, these effects were attenuated by the B co-treatment (compared with the I/R or I/R + DTT group, Fig. 4). Although DTT alone up-regulated the GRP78, ATF6 and CHOP levels, but treated with B together have a decrease effect of those molecular. (Fig. 4).

### Exogenous bisoprolol treatment significantly attenuated the apoptosis and inflammation in myocardial cells caused by myocardial I/R injury

**Figure 5:**
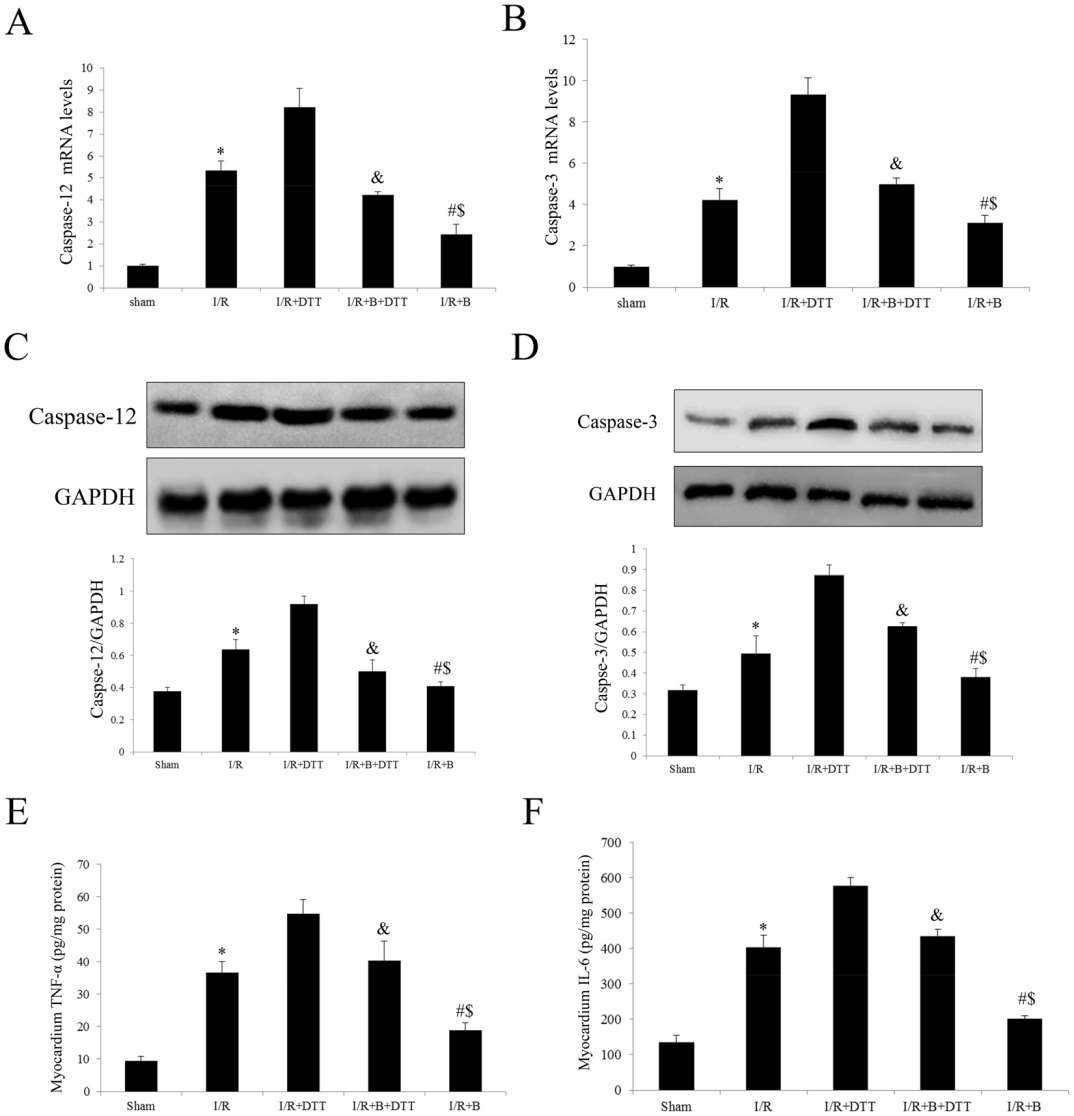
Effect of DTT or B treatment on the expression of Caspase-12 and Caspase-3 after I/R injury in rats. (**A** and **B**) Analysis the transcript levels of Caspase-12 or Caspse-3 by qRT-PCR. (**C** and **D**) Representative Western blotting illustrating the expression of Caspase-12 or Caspase-3 and GAPDH and the quantitative analysis the protein expression level after normalization to GAPDH. (E and F) Effect of pretreat with DTT and B on plasma LDH and CK-MB activities. The results are expressed as the mean ± SEM. n = 6/group. *P < 0.01 vs the sham group; ^#^P < 0.01 vs the I/R group; ^&^P < 0.01 vs the I/R + DTT group; ^$^P < 0.01 vs the I/R + B + DTT group.

To further understand the molecular mechanism of B’s cardioprotective action, we investigated the expression of caspase family molecules in myocardial. As shown in Fig. 5A–D, both in transcript and protein levels, the B treatment significantly decreased the expression of caspase-12 and caspase-3 compared with I/R group. In the I/R + B + DTT group, the B’s reduce effect was abolished compared with I/R + B group. In addition, we measured the expression of IL-6 and TNFα in myocardial tissue homogenate. As shown in Fig. 5E and F, the myocardial I/R injury significantly up-regulated the expression, but this was markedly down-regulated by the B treatment. However, all the protective effects of B were abolished by the DTT co-treatment (compared with the I/R + B group, P < 0.01, Fig. 5E,F).

### Bisoprolol significantly inhibited the UPR and inflammation response in the simulate ischemia reperfusion (SIR) injured H9C2 cells, where these effects can abolish by DTT co-treatment

**Figure 6:**
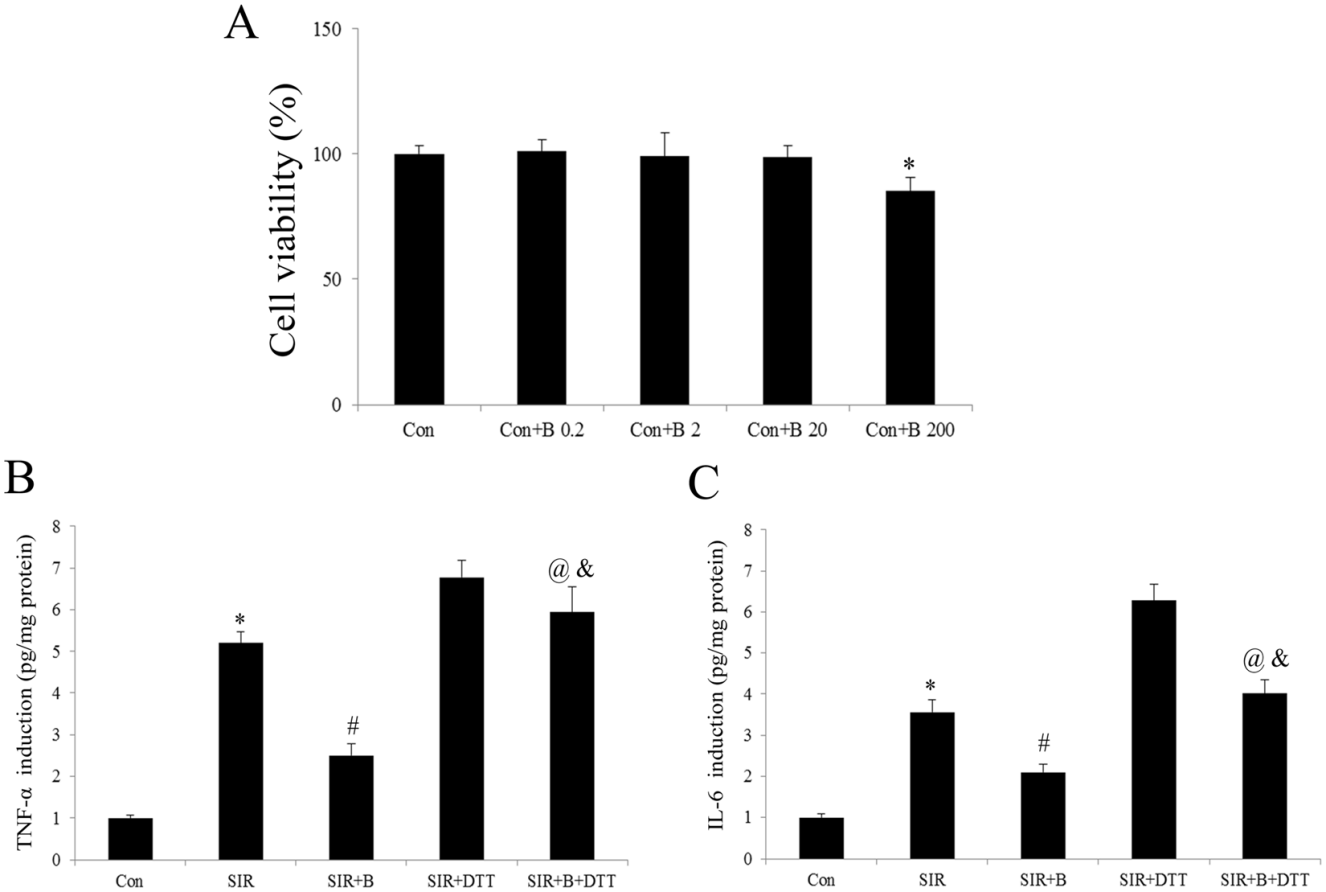
In the cultured H9C2 cells subjected to SIR treatment, B significantly reduced the inflammation response, whereas DTT markedly blocked these effects. (**A**).Viability of H9C2 cells was determined by MTT and was calculated by dividing the optional density of bisoprolol by the optical density of Sham control. (**B**) Cell supernatants TNF-α level. (**C**) Cell supernatants IL-6 level. The results are expressed as the mean ± SEM of 6 separate experiments. *P < 0.01 vs the Con group; ^#^P < 0.01 vs the SIR group. ^@^P < 0.01 vs the SIR + B group; ^&^P < 0.01 vs the SIR + DTT group.

**Figure 7:**
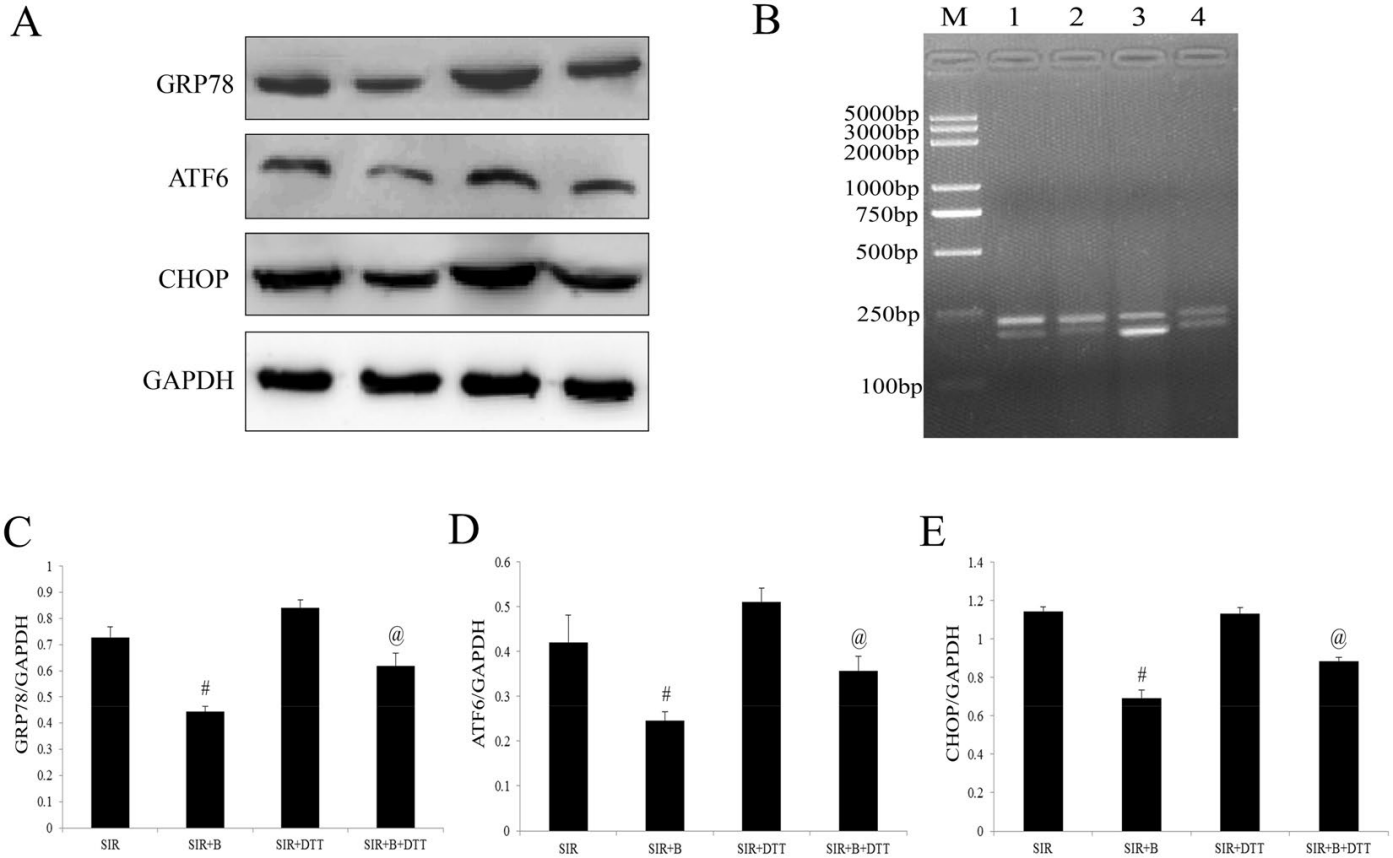
Bisoprolol down-regulated UPR signaling whereas DTT treatment blocked these effects. Normal H9C2 cells pretreated with B or DTT then subjected to the SIR. (**A**) Representative western blots. (**B**) Analysis the effect of XBP1 processing by RT-PCR in H9C2 cells. (**C**) GRP78/GAPDH ratio. (**D**) ATF6/GAPDH ratio. (**E**) CHOP/GAPDH ratio. The results are expressed as the mean ± SEM of 6 separate experiments with triplicate samples for each experimental condition within each experiment. ^#^P < 0.01 vs the SIR group. ^@^P < 0.01 vs the SIR + B group.

The *in vitro* experimental results were consistent with those obtained from the myocardial I/R-injured rat hearts. The H9C2 cells were treated with B at 0.2, 2, 20, or 200 mmol/L for 24 h, and then performed the cell viability by MTT assay. Compared with the control group, no significant cell viability changes were found in the 0.2, 2 and 20 mmol/L groups (P > 0.05, Fig. 6A). However, the 200 mmol/L B treatment markedly affected cell survival (P < 0.01, compared with the Con group, Fig. 6A). Therefore, the 20 mmol/L B dose was chosen for the present study. Firstly, we measured cardiomyocyte inflammation markers in the cultured H9C2 cells. As depicted in Fig. 6B and C, shown TNF-α and IL-6 levels were both markedly decreased by B treatment (P < 0.01, compared with the SIR group). As expected, DTT co-treatment blocked these effects by greatly upregulated TNF-α and IL-6 levels compared with SIR + B group. Then we investigated the cellular UPR signaling pathway in the B or DTT treatment H9C2 cells. Figure 7 showed that B markedly down-regulated the GRP78, ATF6 and CHOP in protein level in the SIR + B group (compared with the SIR group, P < 0.01). Although DTT alone up-regulated the GRP78, ATF6 and CHOP levels, but treated with B together have a decrease effect of those molecular. (Fig. 7A,C–E). The splice effect of XBP in SIR and SIR + DTT group was activated which detected by RT-PCR (Fig. 7B). However, these effects were attenuated by the B co-treatment (compared with the SIR or SIR + DTT group, Fig. 7B).

### Bisoprolol protected the H9C2 cells from the SIR-induced apoptosis, but these effects were abolished by DTT co-treatment and CHOP overexpression

**Figure 8:**
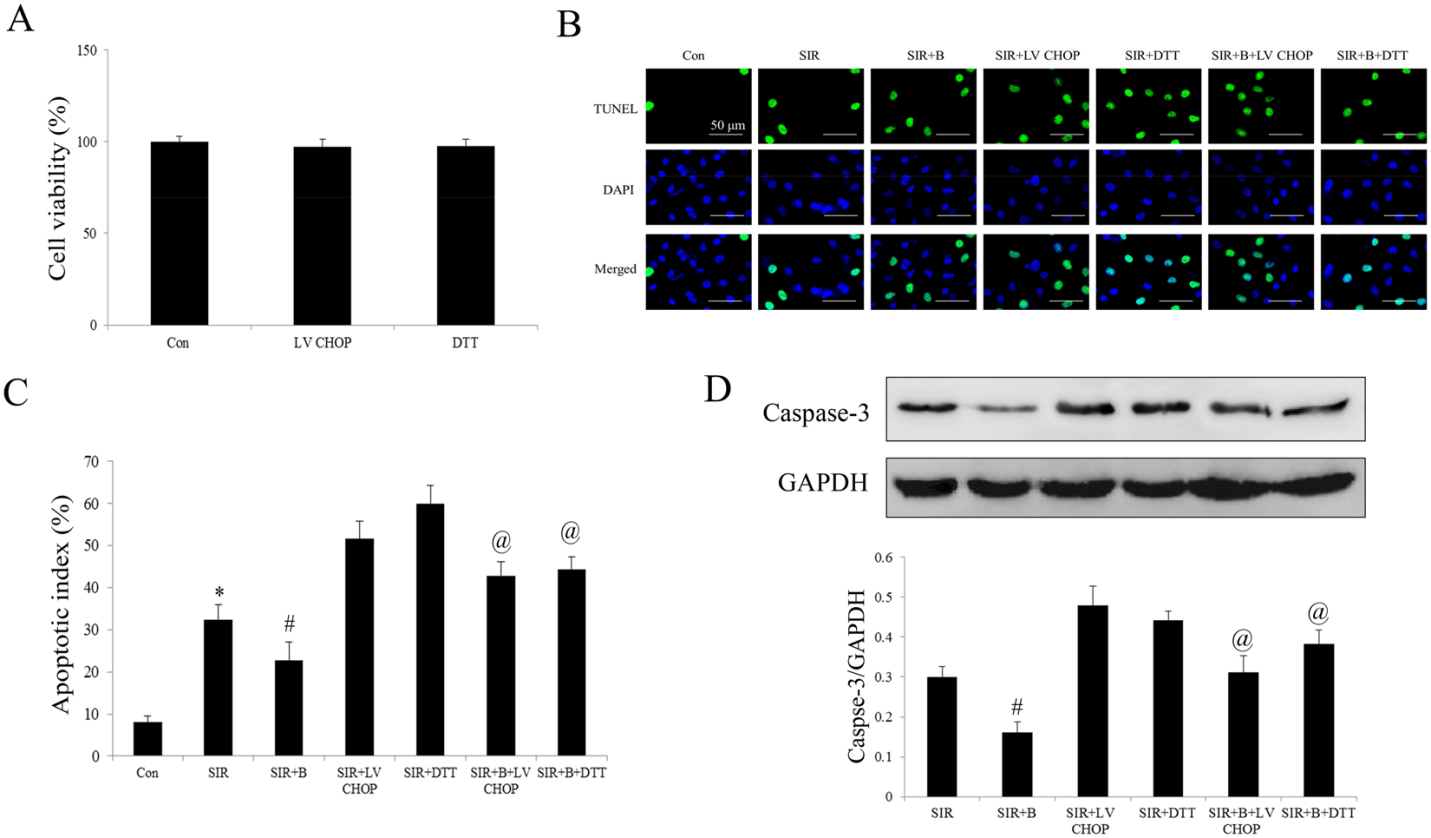
Effects of bisoprolol and overexpression CHOP on cell viability and the apoptotic index in SIR-injured H9C2 cells. (**A**) Viability of H9C2 cells determined by MTT. (**B**) Representative photomicrographs of *in situ* detection of apoptotic cardiomyocytes by TUNEL staining. Green fluorescence shows TUNEL-positive nuclei; blue fluorescence shows nuclei of total cardiomyocytes, original magnification × 400. (**C**) Percentage of TUNEL-positive nuclei. (**D**) Representative Western blotting illustrating the expression of Caspase-3 and GAPDH and the quantitative analysis the protein expression level after normalization to GAPDH. The results are expressed as the mean ± SEM of 6 separate experiments. *P < 0.01 vs the Con group; ^#^P < 0.01 vs the SIR group. ^@^P < 0.01 vs the SIR + B group.

As the Fig. 8A shown, the H9C2 cells were treated LV CHOP or DTT, the cell viability was no significant changes compared with Con group. Second, we found that the B treatment effectively increased the cell viability following SIR by TUNEL assay (compared with SIR group, P < 0.01, Fig. 8B and C). However, the protective action of B was abolished by treatment with the DTT or overexpression of CHOP mediated by lentivirus (compared with SIR + B group, P < 0.05). Meanwhile, we measured the expression of caspase-3 in the different treat group, as shown in Fig. 8D, the suppression effect of caspase-3 expression by B was abolished by DTT or overexpression of CHOP.

## Discussion

In the present study, we utilized both *in vivo* and vitro models to investigate the protective effect of bisoprolol against myocardial I/R-induced cardiac damage. We found that bisoprolol markedly ameliorated the myocardial I/R injury by reducing the UPR signal pathway. Importantly, UPR signaling was proven to play a critical role in this process. This study could help to understand the pharmacology of bisoprolol in the treatment of ischemic heart disease and promote the development of an understanding mechanism of I/R injury.

The benefits of β blocker therapy in the treatment of heart failure are universally accepted. It is well understood that beta-blocker therapy can reduce the morbidity and mortality in heart failure patients^26,27^. With advances in the management of heart disease and the widespread recognition of their effects, beta-blockers are the abundant use of the current treatment for heart failure. However, the mechanism of the protective effects of the beta-blockers is rarely been studied.

In the current study, both *in vivo* and *in vitro* experiments showed that bisoprolol ameliorated myocardial I/R injury and proved that bisoprolol treatment induced a significant decrease of the molecules of UPR expression and reduced the inflammation response. The inhibit UPR signaling was also associated with a decrease of Caspase-12 and Caspase-3 expression. As expected, these effects were both abolished by UPR activator DTT and overexpression of CHOP expression. These results all suggest that the cardioprotection of bisoprolol treatment involves the inhibition of UPR signaling, and decrease the inflammation response, Caspase-12 and Caspase-3 expression. Interestingly, we found in bisoprolol treated animals or cells significantly reduced myocardial IL-6 accumulation and TNF-𝛼 production, which indicated that bisoprolol also ameliorated cardiac inflammation.

Furthermore, the present study was carried out to elucidate the underlying mechanisms of bisoprolol’s cardioprotective action by attenuate UPR signaling. It has been proven that excessive ER stress participates in ischemia/reperfusion injury^28,29^. Of the many signaling pathways involved in ER stress, PERK/eIF2α/ATF4, one of the UPR signal pathway has been found to contribute strongly to programmed cell death during myocardial I/R injury^30^. Intriguingly, in the present study, we found that bisoprolol down-regulate all the three pathway of the UPR signal pathway, and treatment with DTT and overexpression CHOP can abolish the effect of bisoprolol. Furthermore, bisoprolol reduced the pro-inflammatory cytokine which induced by ER stress and significantly decreased the LDH and CK-MB levels both *in vivo* or vitro. There was study found that the ER stress pathway involving CHOP was activated and played a role in the pathogenesis of septic organ injury^31^. And in septic mice that CHOP inhibition effectively decreased the organ damage and increased the overall survival rate^32^. Thus, it is worthwhile to further investigate bisoprolol’s potent therapeutic effect on myocardial I/R injury with a focus on CHOP.

So far, several clinical investigations have established that there is a beneficial effect of bisoprolol treatment on the cardiovascular system in patients with high-risk patients after successful major cardiac vascular surgery^25,33,34^. Hence, bisoprolol was suggested to be a safe and effective option for patients under conditions of moderate cardiovascular risk. Based on our data, ameliorate the myocardial I/R injury by bisoprolol pretreatment is correlate with UPR signaling pathway in rats. The main novel finding of this study is to demonstrate a cardioprotective effect of treatment with bisoprolol via inhibition of UPR and the stress-associated intracellular factors. In addition, mitochondrial apoptosis pathway is another important target of cardioprotective signalling. There have many researches pointed out that myocardial hypoxia is closely associated with cardiac dysfunctions, in particular myocardial infarction (MI), ischemia reperfusion injury, and hypertrophy^35^. So the UPR is merely a part of participate in regulate the myocardial I/R injury. Further clinical studies might be needed before a conclusion can be reached on the utility of bisoprolol in this setting.

Taken together, we demonstrated that bisoprolol reduced myocardial I/R injury via inhibiting of the UPR signaling and inflammation response. These findings may provide new mechanistic insight into the cardioprotective effects of bisoprolol.

## Materials and Methods

### Antibodies and reagents

Evans blue dye, 2,3,5-triphenyltetrazolium chloride and Dithiothreitol (DTT) obtained from sigma Aldrich, USA. Antibodies for GRP78, CHOP, ATF6, XBP1 and GAPDH were obtained from Santa Cruz Biotechnology, USA; Antibody for Caspase-12 and Caspase-3 were obtained from Sigma Aldrich, USA. The secondary antibody HRP-labeled Goat Anti-Mouse IgG and Goat Anti-Rabbit IgG were obtained from beyotime, China.

### Creation of myocardial I/R rat model ***in vivo***

Adult male Sprague-Dawley (SD) rats (250–300 g) were procured from the Animal Center of Jinling Hospital (Nanjing, China). Animal maintenance and experimental procedures were conducted after approved by the Animal Care Committee of Nanjing University (Nanjing, China) and in accordance with the strict guidelines of the Institutional Animal Ethics Committee (IAEC). SD rats were treated with B (10 mg·kg^−1^·d^−1^) for 1 week, in the absence or presence of the injected UPR activator dithiothreitol (DTT, 100 umol·kg^−1^) before ischemia 10 minutes and then subjected to myocardial I/R surgery. The rats were anaesthetized using pentobarbital sodium (3%) via intraperitoneal injection. The surgical procedures were performed as previously described^36^. Briefly, after pericardiotomy, a 6–0 silk suture was placed under the left coronary artery (LCA), which was occluded by tightening the snare using a lightweight hemostatic clamp. After 30 min of myocardial ischemia, the suture was loosened and the myocardial was reperfused for 6 hr (for analysis transcript and protein levels and for quantification of myocardial apoptosis and infarct size), for 72 hr (for cardiac function determination). The sham-operated animals underwent the same surgical procedures except that the suture around the LCA was not fastened. Ischemia was confirmed by a transient decrease in blood pressure and cyanosis on the myocardial surface. Reperfusion was indicated by an epicardial hyperemic response and the rapid disappearance of cyanosis.

### Identification of myocardial infarct size in rats with I/R

At the corresponding time of the experiment, the rats were anaesthetized and the LCA was reoccluded, injected with 2% Evans blue dye via the external jugular vein, then the area at risk (AAR) in the heart can be displayed. About 5 min later, the heart was rapidly excised and frozen at −80 °C approximately 10 min. After that, the frozen heart was cut into 5 transverse slices of equal thickness. Then the slices were incubated in phosphate-buffered 1% 2,3,5-triphenyltetrazolium chloride (TTC) for 10 min at 37 °C. In the end, the slices fixed with 4% paraformaldehyde prepared in PBS (pH-7.4). The AAR means the area that was not stained with Evans blue dye. In the AAR area, where not stained by TTC were confirmed as the area of infarction (AI). The AAR, AI and ventricle size (VS) were assessed by using Image-J software^37^.

### Measurement of LDH and CK activities in rats with I/R

After the end of the reperfusion, plasma samples (1 mL) were obtained from carotid aorta using a heparinized syringe and immediately centrifuged. The activities of lactate dehydrogenase (LDH) and creatine kinase MB (CK-MB) were assayed using spectrophotometrically (Beckman DU 640, Fullerton, CA, USA) in a blinded manner as described before^38^, which purchased from the Institute of Jiancheng Bioengineering (Nanjing, China).

### Apoptotic Cell Assay

For detection of DNA fragmentation, terminal deoxynu-cleotidyl transferase (TdT) -mediated dUTP nick end labeling (TUNEL) assay was performed as described previously^39^. Briefly, After *in vivo* I/R procedure, the hearts were fixed in 10% buffered formalin and subsequently embedded in paraffin to obtain 5 μm-thick sections. DeadEnd™ Fluorometric TUNEL system (Promega) was used to stain for apoptotic nuclei. The number of TUNEL-positive cells was expressed as a percentage of total cells. TUNEL-positive nuclei were counted by randomly selecting 10 fields of the mid ventricular section. Nuclei were stained by 4′, 6′-diamino-2-phenylen-dolehydrochloride (DAPI) (Invitrogen, USA) and then visualized under fluorescence microscope (Leica DM5000 B, Leica Microsystems). Six specimens were employed for apoptotic index analysis. The apoptotic index was calculated as the ratio of the number of TUNEL-positive neurons to the total number of nuclei.

### Echocardiography

Six rats from each group were used for cardiac function determination by Doppler echocardiography with a 15 MHz linear transducer (Visual Sonics Vevo 2100, Canada). To avoid interference in the acoustic signal by residual air trapped inside the chest cavity, echocardiography was conducted after 72 h of reperfusion, by which time most of the residual air had been absorbed. Baseline echocardiography was obtained 30 min before surgery. M-mode echocardiography was used to evaluate the cardiac dimensions and function. Left ventricular ejection fraction (LVEF) and left ventricular fractional shortening (LVFS) were calculated with computerized algorithms.

### Detection of IL-6 and TNF-α Level

After reperfusion, the levels of IL-6, TNF-𝛼 in cardiomyocytes supernatant, and myocardial tissue homogenate were detected in strict accordance with manufacturer’s instructions^40,41^. BCA kit was used to detect the protein quantization.

### Reverse Transcription-Polymerase Chain Reaction (RT-PCR)

For X-box binding protein (XBP1) analysis, cDNA was synthesized as previous mentioned. PCR was performed using Geneamp PCR system (Singma) with Taq DNA polymerase (CWBIO, China). The primer specific for target sequences of XBP1: forward, GAGCAGCAAGTGGT GGAT; reverse, AGGCAACAGCGTCAGAAT. Amplification conditions included: 94 °C for 5 min; 30 cycles at 94 °C for 30 s, 58 °C for 30 s and 72 °C for 45 s; 72 °C for 5 min. When UPR is induced, XBP1mRNA is spliced and activated through activation of the ER stress sensor IRE1^42^. The primer sets for XBP1 were amplified the PCR products of 297 bp for the unspliced form and 271 bp for the spliced form. The amplified RT-PCR products were separated by 3% agarose gel electrophoresis and visualized with ethidium bromide staining under UV light.

### Cell culture and cell viability measurement

H9C2 cells were cultured in high glucose Dulbecco’s-modified Eagle’s medium (DMEM; GIBCO, UK) containing 2 mM L-glutamine, 1.5 g/l sodium bicarbonate, 4.5 g/l glucose, 10 mM HEPES, 1.0 mM sodium pyruvate, 0.1 mM nonessential amino acids and 10% fetal bovine serum (FBS) (GIBICO, UK). Cultured H9C2 cardiomyocytes pretreated with B (2 μM, 48 h before simulate ischemia) or DTT (2 mM, 3 h before simulate ischemia), then subjected to SIR injury. The H9C2 cells, 1 × 10^4^per well, were seeded in 96-well culture plates. After different treatment, the SIR was performed. The cell viability was measured using the 3-(4, 5-dimethylthiazol-2-yl)-2, 5-diphenyltetrazolium bromide (MTT) assay as described previously^43^. Briefly, after the cells were treated and washed with PBS, 10 μL of MTT dye was added to each well at a final concentration of 0.5 mg/mL. After 4 h of reperfusion, 100 μL of DMSO was added to dissolve the formazan crystals. The absorbance was measured using a microtiter plate reader (SpectraMax 190, Molecular Device, USA) at a wavelength of 570 nm. The cell viability was calculated by dividing the optical density of samples by the optical density of the control group.

### Simulate ischemia reperfusion (SIR) treatment

The SIR treatment was performed using physiological concentrations of potassium, hydrogen, and lactate. The procedure was performed as described previously^44^. Briefly, the H9C2 cells were exposed to an ischemic buffer containing (in mmol/L) 137 NaCl, 12 KCl, 0.49 MgCl_2_, 0.9 CaCl_2_, and 4 HEPE S. This buffer was also supplemented with (in mmol/L) 10 deoxy-glucose, 0.75 sodium dithionate, and 20 lactate. The buffer pH was 6.5. Stimulated ischemia was the procedure that the cardiomyocytes were incubated for 2 h in a humidified cell culture incubator (1% O_2_, 5% CO_2_, 96% N_2_, 37 °C). Reperfusion was performed by returning the cells to normal culture medium for 4 h in a humidified cell culture incubator (21% O_2_, 5% CO_2_, 37 °C).

### Lentivectors construction and lentivirus production

The primers used to over-express CHOP to generate pCDH-CMV-CHOP contained EcoRI and BamHI restriction sites, shown as forward: TGCTCTAGAATGGCAGCTGAGTCTCTG, reverse: CGGGATCCTCATGCTTGGTGCAGACTG. The CHOP gene was cloned into the over-expression lentivector pCDH-CMV-MCS-EF1- GreenPuro (CD513B-1) (SBI, Mountain View, CA, USA) to generate pCDH-CMV-CHOP. HEK-293T cells were transfected with the construct along with three other plasmids (pGag/Pol, pRev, pVSV-G) into HEK293T cells using TurboFect (Thermo scientific) according to the manufacturer’s protocol. The overexpression experiment was performed as follows. H9C2 cells (4 × 10^6^/well) were seeded in a 6-well plate. After 24 h, 50% of the medium in each well was removed, and polybrene was added to a final concentration of 8 μg/ml. The CHOP overexpressing lentiviruses (MOI = 1) were added to the cells. After an overnight incubation at 37 °C, the medium was replaced with fresh medium and incubated for another 72 h. The cells were then used to evaluate the expression of CHOP or for further experiments.

### Quantitative Real-Time PCR (qRT-PCR)

At the indicated times after reperfusion, hearts were removed and frozen in liquid nitrogen immediately then stored at −80 °C. Total RNA was isolated from hearts using TRIZOL reagent (Invitrogen) according to manufacturer’s protocol. Complementary DNA (cDNA) was synthesized using the Primescript^TM^ RT reagent kit (Takara Bio, Dalian, China) according to the manufacturer’s instructions. Three-step quantitative real-time PCR was performed using the Thermal Cycler Dice^R^ Real Time System with SYBR ExScriptTM RT-PCR Kit (Takara Bio, Dalian, China). The primers used for target sequences of GRP78, ATF6, CHOP, Caspase-12 and GAPDH as following: GRP78: forward, ACTGGAATC CCTCCTGCTC; reverse, CAAACTTCTCGGCGTCAT; ATF6: forward, GCAGGTGTATTACGCTTCG; reverse, TTCGGTCTTGTGGTCTTG TT; CHOP: forward, CACAAGCACCTCCCAAAG; reverse, CCTGCTCCTTCTCCTTCAT; Caspase-12, forward: GGTCTTTATGTC CCACG; reverse, CAGTATGTCTGCCTCTGC; Caspase-3, forward: GGACTGCGGTATTGAGA; reverse, CGGGTGCGGTAGAGTA; GAPDH: forward, GCAAGTTCAACGGCACAG; reverse, GCCAGTAGACTCCACGACAT. The amplification conditions was performed at 95 °C for 10 minutes, followed by 40 cycles at 95 °C for 10 seconds, 58 °C for 15 seconds and 72 °C for 15 seconds. Levels of target gene mRNA were normalized to those of reference genes GAPDH.

### Western Blotting Analysis

Hearts were homogenized in five volumes of ice cold lysis buffer (200 mmol/l HEPES (pH 7.4), 250 mmol/l sucrose, 1 mmol/l dithiothreitol, 1.5 mmol/l MgCl_2_, 10 mmol/l KCl, 1 mmol/l EDTA, 1 mmol/l EGTA, 0.1 mmol/l PMSF, and 1% protease and phosphatase inhibitor cocktails (Sigma)). The lysates homogenates were spun at 12,000 g for 5 min and the supernatant was used. For SDS-PAGE analysis an equal amount of protein (50 μg) was loaded in each well and subjected to 10% sodium dodecyl sulfate-polyacrylamide gel electrophoresis. The separated proteins were then transferred onto PVDF membrane (Millipore, MA, USA) and blocked in 5% non-fat dry milk prepared in 1 × TBS. The membranes were incubated with the primary antibodies for 4 h at RT or overnight at 4 °C (dilutions 1:500-1:1,000). The following primary antibodies were used: GRP78, ATF6, CHOP, XBP1, Caspase-12, Caspase-3 and GAPDH. After washing the membranes were incubated with secondary antibodies (1:5000) for 2 h at RT. The secondary antibodies used were horseradish peroxidase (HRP) conjugated anti-IgG corresponding to the primary antibodies. The blots were developed by using ECL Plus detection system (Amersham Pharmacis). The relative band intensity was measured by Image J software.

### Statistical Analysis

Significant differences between sham and I/R groups were made using ANOVA followed by Newman-Keuls multiple comparison test. The statistical analyses were performed using a 2-tailed Student’s t-test and represented as mean ± SEM. *P < 0.05 and **P < 0.01 were considered statistically significant.
